# Is Zebrafish a Good Model for the Alpha‐Gal Syndrome?

**DOI:** 10.1096/fj.202500687R

**Published:** 2025-05-02

**Authors:** Rita Vaz‐Rodrigues, José de la Fuente

**Affiliations:** ^1^ SaBio, Instituto de Investigación en Recursos Cinegéticos IREC‐CSIC‐UCLM‐JCCM Ciudad Real Spain; ^2^ Department of Veterinary Pathobiology, College of Veterinary Medicine Oklahoma State University Stillwater Oklahoma USA

**Keywords:** allergy, alpha‐gal syndrome, animal model, biomedicine, tick, zebrafish

## Abstract

The alpha‐Gal syndrome (AGS) is an underdiagnosed tick‐borne allergy characterized by both immediate and delayed IgE‐mediated anaphylactic reactions to the galactose‐alpha‐1,3‐galactose (alpha‐Gal) epitope. Common manifestations include gastrointestinal, cutaneous, and respiratory symptoms appearing 2–6 h after the consumption of mammalian meat or derived products. Zebrafish ( *Danio rerio* ) are emerging as essential animal models in biomedical studies, due to their anatomical, genetic, and physiological similarities to humans, with significant applications in toxicology, behavioral research, oncology, and inflammation studies. The mechanisms associated with AGS are sustained by studies in the humanized α1,3GalT‐KO C57BL/6 mouse ( *Mus musculus* ) and zebrafish animal models for the production of anti‐alpha‐Gal antibodies in response to tick saliva, the development of allergic reactions in animals sensitized with tick protein extracts following mammalian meat consumption, and the identification of immune mechanisms. The immune mechanisms characterized in both models are associated with a skewed type 2 immune response, triggering Toll‐Like receptor (TLR) signaling pathways, IL‐4 production, and humoral activity. These results support the use of both models rather than a single one for a more comprehensive characterization of AGS‐associated immune mechanisms. In this study, we focused on the use of zebrafish as a model for biomedicine research in immunity, infectious, and allergic diseases, with a particular emphasis on the AGS and the identification of candidate therapeutic interventions. Based on insights from multiple studies, we concluded that zebrafish is a suitable model for studying the AGS, considering the addressed limitations and in combination with the α1,3GalT‐KO mouse model.

## 1 Ticks and the Alpha‐Gal Syndrome

Ticks are ectoparasite vectors of pathogens affecting human and animal health worldwide [1]. After a bite and during blood feeding, tick saliva modulates the host immune response that may be associated in some tick species with allergic reactions such as the alpha‐Gal syndrome (AGS) [2, 3, 4, 5]. During hominid evolution, events of catastrophic selection resulted in the inability to synthesize certain molecules such as the oligosaccharide galactose‐alpha‐1,3‐galactose (alpha‐Gal) [6, 7]. This evolutionary adaptation resulted in the protective effect of naturally produced anti‐alpha‐Gal antibodies in response to gut microbiota, pathogens, or ectoparasites with this modification, while also contributing to the adverse effects of the AGS [6, 8, 9].

The AGS is associated with both immediate and delayed pathognomonic anaphylactic allergic reactions, mediated by immunoglobulin E (IgE) against alpha‐Gal epitopes [10, 11]. Individuals may also develop gastrointestinal symptoms (reflux, emesis, and diarrhea), cutaneous signs (pruritus, urticaria, and angioedema), and respiratory issues (dyspnea and hypoxia) [12, 13]. These reactions typically occur 2–6 h after the consumption of mammalian meat or derived products such as dairy, heparin, and gelatins [9, 14]. The alpha‐Gal modifications are present in glycoproteins and glycolipids from the saliva of specific tick species, as well as in the cells and secretions of non‐catarrhine mammals [15, 16, 17, 18]. Recently, reactive proteins in patients with AGS were identified in bovine milk [19]. Additionally, the AGS has been linked to other biomolecules, including the inflammatory mediator prostaglandin E2 (PGE2) [20, 21, 22].

Food allergies, including AGS, are a growing worldwide concern, with rising prevalence rates and increased healthcare utilization [23, 24]. The prevalence and incidence of AGS cases have been more thoroughly characterized in the United States (US). A total of 90 018 (30.5%) individuals tested positive for alpha‐Gal‐specific IgE (sIgE) antibodies between 2017 and 2022, with the number of positive results increasing from 13 371 in 2017 to 18 885 in 2021 [25]. Additional research provided similar insights into the pattern of AGS seroprevalence in the United States [26]. In several other countries such as Mexico, Spain, France, Germany, and Sweden, the prevalence of AGS cases is also increasing [13, 23, 27]. Nevertheless, limited awareness among healthcare professionals and the general population, insufficient diagnostic capacity, and a lack of standardization in diagnostic practices still lead to delayed diagnosis, misdiagnosis, and underdiagnosis of AGS [28, 29, 30, 31]. The diagnosis of AGS requires considering multiple factors, including clinical history, positive alpha‐Gal sIgE blood testing, case‐to‐case variable symptomatology, and improvement on a mammalian meat‐restricted diet [13, 32, 33]. Additionally, it is important to consider that anti‐alpha‐Gal antibodies may also be related to other diseases, such as Lyme borreliosis [34], coronavirus disease (COVID‐19) [35], and Guillain‐Barré syndrome [36]. Disease management remains limited, focusing on avoiding tick bites and allergenic foods, such as mammalian meats and byproducts, along with the acute management of IgE‐mediated anaphylactic reactions [37, 38]. Additional information is disclosed at the Alpha‐Gal Information (AGI) website [39].

**FIGURE 1:**
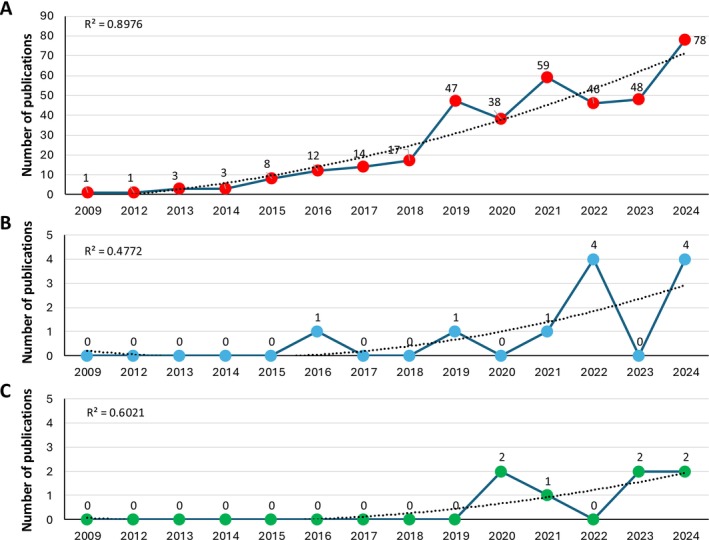
Bibliometric analysis of alpha‐Gal syndrome (AGS) entries. Bibliometric analysis was conducted in the PubMed database [40] on January 8th 2025, to include all publications in 2024 with search queries (A) “alpha gal syndrome”, (B) “alpha gal syndrome mouse” (review papers excluded), and (C) “alpha gal syndrome zebrafish” (review papers excluded and alpha‐Gal protective capacity included). Polynomial regression was applied to predict the growth publication tendency, with the polynomial correlation coefficient (*R* ^2^) presented for each dataset.

Research on AGS has increased in recent years (Figure 1) to face the challenges and limitations associated with diagnosis, management, and prevention strategies [32, 38, 41]. Despite these advances, the molecular mechanisms driving the wide variety of AGS‐related symptoms in individuals exposed to tick bites have yet to be fully characterized [10, 21, 42]. Several studies indicate that patients diagnosed with AGS exhibit differential recognition of tick proteins [43, 44] and that various biomolecules in the tick sialome can trigger the host humoral response to alpha‐Gal [45, 46, 47]. Accordingly, tick proteins and lipids with alpha‐Gal modifications present immunological regulatory functions, and salivary glycans are potentially involved in the development of host acquired tick resistance [5, 18]. Additionally, biomolecules with the glycan moiety alpha‐Gal may have a role in tick cement formation [48].

Animal models are essential to better understand disease pathogenesis and to address key questions and overcome limitations regarding AGS molecular mechanisms. For this purpose, the α1,3GalT‐KO mouse model ( *Mus musculus* ) on the C57BL/6 background was genetically modified to knockout the α1,3‐galactosyltransferase (α1,3GalT) gene, resulting in the inability to produce alpha‐Gal epitopes and thus mimicking human biology [49, 50, 51]. Results employing this animal model provided further insights into the potential molecular mechanisms associated with the AGS (Table 1). Moreover, α1,3GalT‐KO mice have also been used to study the effects of alpha‐Gal nanoparticles in regenerating skin wounds, heart, and spinal cord injuries, as well as to assess the impact of immune‐mediated reactions to xenoantigens [62, 63].

**TABLE 1:** The α1,3GalT‐KO C57BL/6 animal model ( *Mus musculus* ) and associated results for the alpha‐Gal syndrome (AGS) studies, including all publications from 2024 and prior.

Results from α1,3GalT‐KO mice in AGS studies	References
Induction of both IgG and IgE anti‐alpha‐Gal antibodies in response to *Amblyomma sculptum* tick feeding exposure or injected tick saliva	Araujo et al. [52]
Production of total and tick‐specific IgEs in response to *Amblyomma americanum* tick protein extracts, followed by increased sIgE levels to alpha‐Gal and hypersensitivity reactions in sensitized animals after oral administration of mammalian meat. The IgE response was dependent on cognate CD4^+^ T cells and B cell‐intrinsic Toll‐like receptor (TLR) adaptor protein MyD88	Chandrasekhar et al. [53]
Elevated sIgE levels to alpha‐Gal and development of characteristic allergic reactions to pork fat and pork kidney following sensitization with *A. americanum* tick salivary gland extract	Choudhary et al. [54]
Total and alpha‐Gal specific IgEs antibodies in response to alpha‐Gal conjugated to human or mouse serum, blood feeding and partially fed and unfed *A. americanum* ticks functioning as sensitizers. The “transmission” hypothesis referred to tick‐mediated transmission of the alpha‐Gal sensitizer acquired from non‐human blood to humans in the intrastadial host feeding switch was not supported	Maldonado‐Ruiz et al. [55]
A proposed immunotherapy based on poly‐L‐lysine‐based alpha‐Gal‐glycoconjugates for potential treatment of allergic disorders like AGS by selectively inhibiting the production of anti‐alpha‐Gal IgE antibodies	Olivera‐Ardid et al. [56]
The analysis of anti‐alpha‐Gal antibodies in a glycan complex, via X‐ray crystallographic, revealed a common binding motif shared by human and α1,3GalT‐KO mouse antibodies. This motif consisted of a germline‐encoded tryptophan residue at Kabat position 33 (W33) within the complementarity‐determining region of the variable heavy chain (CDRH1). The germline‐encoded W33 residue was found to be critical for antigen binding	Langley et al. [57]
A novel high‐affinity, alpha‐Gal epitope‐specific monoclonal IgG1 antibody (27H8) was produced in α1,3GalT‐KO mice. This antibody did not react with alpha‐Gal positive intestinal bacteria, raising questions about whether commensal bacteria express the native alpha‐Gal epitope	Kreft et al. [58]
Alpha‐Gal‐sensitized α1,3GalT‐KO mice displayed increased levels of alpha‐Gal‐specific IgE and IgG1 antibodies, along with a higher number of basophils at the sensitization site, as well as alpha‐Gal‐specific B cells, germinal center B cells, and B cells of the IgE and IgG1 isotypes in skin‐draining lymph nodes. Furthermore, the murine model developed systemic anaphylaxis, dependent on interleukin‐4 (IL‐4), when challenged with alpha‐Gal‐containing glycoproteins and glycolipids	Hils et al. [59]
Higher total IgE and IgG1 and anti‐alpha‐Gal IgG1antibody levels in response to sensitization with *A. americanum* in comparison with *Amblyomma maculatum* nymphs and followed by pork meat challenge. After *A. americanum* infestation and pork meat challenge, mouse immune response shifted towards Th2 and facilitated host sensitization to alpha‐Gal. AGS may be associated with specific tick species	Sharma et al. [60]
Prophylactic immunotherapy using biodegradable nanoparticles with encapsulated alpha‐Gal prior to tick protein‐induced alpha‐Gal IgE sensitization activates Th2 cytokines and suppresses the production of alpha‐Gal sIgE associated with hypersensitivity reactions to enhance immune tolerance against alpha‐Gal sensitization. Accordingly, alpha‐Gal‐containing nanoparticles are a potential strategy for increasing alpha‐Gal‐specific immune tolerance through modifications of immune mechanisms associated with alpha‐Gal‐induced IgE‐mediated allergic reactions	Saunders et al. [61]

Additionally, a new humanized mouse model (Hu‐NSG/alpha‐Gal^null^) was designed for preclinical assessments of viral and tumor alpha‐Gal‐based vaccines, xenotransplantation research, and in vivo biomaterials evaluation, with a focus on characterizing specific immune responses [64]. Recently, the well‐established C3H/HeN and BALB/c mouse strains were employed to study the effects of *Ixodes ricinus* tick bites on the skin, as well as intestinal changes that could potentially contribute to a predisposition to red meat allergy [65]. Results revealed that *I. ricinus* bites triggered epidermal hyperplasia, spongiosis, and the accumulation of eosinophils and mast cells in the bitten skin, while also promoting the upregulation of genes associated with pathways activated by mechanical skin injury. Moreover, *I. ricinus* bites led to an increase in total serum IgE levels and an expansion of tuft cells and mast cells in the jejunum.

## 2 History of the Zebrafish ( *Danio rerio* ) Animal Model in Biomedicine

George Streisinger (University of Oregon, USA) pioneered the use of zebrafish ( *D. rerio* ) model in biomedical research. During the 1970s and 1980s, he developed key techniques for zebrafish breeding, genetic manipulation, and embryo observation [66]. Since then, zebrafish have been established as a powerful animal model for studying molecular genetics and vertebrate embryonic development [67, 68]. Streisinger's work laid the foundation for using zebrafish in several areas of biomedical research, including human and veterinary disease modeling, metabolic disorders, behavioral studies, effect of environmental factors, and drug discovery [68, 69, 70, 71]. The vertebrate immune system uniquely evolved with the somatic rearrangement and mutations of immunoglobulin genes in lymphocytes and the appearance of immune mast cells with a functional role in both innate and adaptive immune responses [72]. The zebrafish is a key model for studying mast cell biology and evolution, providing functional evidence that mast cell functions were conserved from the last common ancestor of fish and mammals [73].

Zebrafish are emerging as essential animal models in biomedical studies, due to their anatomical, genetic, and physiological similarities to humans, with significant applications in toxicology, behavioral research, oncology, and pulmonary inflammation studies [74, 75, 76, 77, 78]. The zebrafish model has been utilized in the study and development of therapeutics for inflammatory and allergic diseases, including atopic dermatitis, asthma, and other CXCR4‐associated conditions [79]. Furthermore, the integration of omics approaches with zebrafish disease modeling contributes to the characterization of molecular mechanisms underlying human diseases [80, 81, 82].

In summary, and according to Larijani et al. [71], zebrafish is a valid and powerful vertebrate model for preclinical in vivo studies due to (a) easy maintenance with low costs; (b) a short life cycle and high fertility; (c) embryo transparency, external development, and rapid growth, within about 5 days after fertilization; (d) similarities between zebrafish and mammals, including humans, in cellular and biological processes, genetics (i.e., 82% of genes involved in human diseases are orthologous [82]), and preservation of some body systems (e.g., gastrointestinal, cardiovascular, musculoskeletal and nervous) and organs (e.g., pancreas and liver) between zebrafish and humans; (e) the ability of small pharmaceuticals and chemical compounds to penetrate the body; (f) the regeneration of certain organs; and (g) the possibility of genetic manipulation. Nevertheless, limitations to be considered when using the zebrafish model include (a) the phylogenetic distance between zebrafish and humans; (b) the ectothermic nature of zebrafish, making them more susceptible to environmental factors compared to endothermic organisms like humans; (c) the absence of certain organs, such as lungs, which limits studies of some human diseases; and (d) the genome duplication in zebrafish that should be accounted for in genome‐associated studies.

## 3 The Zebrafish Model for the AGS

The zebrafish animal model has been used to study diabetes and lipid‐related diseases due to its similarities with mammalian lipid absorption, processing, and metabolism [83]. Additionally, zebrafish can be used to study immunological factors in respiratory diseases [84], inflammasome [85] and serve as an animal model for investigating microbiota function in metabolic and immune disorders [86, 87]. For example, fatty acid residues, in combination with other compounds such as hydrogen peroxide (H_2_O_2_), induce anaphylactoid reactions in zebrafish through mast cell degranulation [88]. Metabolic reprogramming and inflammation induced by fatty acid‐fueled matrix metalloproteinase production have also been documented in zebrafish [89]. Furthermore, it is important to note that, while behavioral syndromes have been reported in wild zebrafish, they have a limited impact on laboratory animals [90].

Mammals, including humans and mice, have the capacity to produce five heavy chain isotypes of immunoglobulins, including IgM, IgD, IgG, IgA, and IgE [91]. In contrast, three Ig isotypes have been identified in bony fish: IgM, IgD, and IgT/Z [92]. Bony fish IgM is the most prevalent antibody in fish plasma and the most conserved in form and function across vertebrates, playing a role in both innate and adaptive immunity in fish [93, 94, 95]. This isotype mediates complement activation, pathogen phagocytosis, and cellular cytotoxicity [96, 97]. Little is known about IgD physiology, although it may stimulate the production of antimicrobial, opsonizing, pro‐inflammatory, and B cell‐activating factors [98]. Finally, zebrafish have gene sequences encoding IgZ/IgT, functionally analogous to IgA in mammals, and the most important immunoglobulin of bony fish mucosal immunity [92, 99]. This isotype acts on gut mucosal responses and extensively populates teleost skin‐associated lymphoid tissue (SALT) alongside B cells [100, 101].

In the 1900s, the analysis of alpha‐Gal epitope expression on nucleated cells of various species revealed the absence of alpha‐Gal epitopes on fibroblasts derived from non‐mammalian vertebrates including fish, amphibians, reptiles, or birds [102]. Zebrafish, which naturally do not produce alpha‐Gal biomolecules [103], were used as an animal model to study allergic reactions [104] and immune mechanisms mediated by tick salivary biomolecules and mammalian meat consumption associated with the AGS [103, 105, 106, 107, 108] (Table 2). Additionally, this small teleost was used to characterize alpha‐Gal‐mediated immunity against tuberculosis by exposing it to *Mycobacterium marinum* , which causes a tuberculosis‐like illness in fish [109, 110, 111] (Table 2).

**TABLE 2:** The zebrafish ( *Danio rerio* ) animal model and results for the study of alpha‐Gal‐mediated immunity in the alpha‐Gal syndrome (AGS) and tuberculosis, including all publications from 2024 and prior.

Results from zebrafish model in alpha‐Gal‐mediated immunity	References
Presence of anti‐alpha‐Gal IgM antibodies, allergic reactions, abnormal behavior, and feeding patterns in response to *Rhipicephalus sanguineus* saliva and salivary biogenic substances following mammalian meat consumption. Allergic reactions were linked to tissue‐specific TLR‐mediated responses in types 1 and 2 T helper cells (Th1 and Th2), with a potential role for basophils in response to tick saliva	Contreras et al. [103]
Protective response against *M. marinum* in the zebrafish model of tuberculosis. The protective immune mechanisms included B‐cell maturation, antibody‐mediated opsonization of mycobacteria, Fc‐receptor (FcR)‐mediated phagocytosis, macrophage response, interference with the alpha‐Gal antagonistic effect of the TLR2/nuclear factor kappa‐light‐chain‐enhancer of activated B cells (NF‐kB)‐mediated immune response, and upregulation of pro‐inflammatory cytokines such as interleukin 1 beta (IL‐1β)	Pacheco et al. [109]
Protective response against mycobacteriosis by probiotic bacteria with high alpha‐Gal content involves mechanisms such as the modification of gut microbiota composition, B‐cell maturation, anti‐alpha‐Gal IgM antibodies‐mediated control of mycobacteria, induced innate immune responses, beneficial effects on nutrient metabolism and reduced oxidative stress	Pacheco et al. [110]
Identification of *I. ricinus* tick salivary proteins, including allergen‐type antigen p23 (UniProt ID: A0A0K8RKR7) and metalloprotease (UniProt ID: A0A0K8RCY8), without alpha‐Gal modifications, which are involved in modulating the immune response against this carbohydrate	Contreras et al. [105]
Multi‐omics data analysis of *I. ricinus* tick saliva identified pathways and molecules that may contribute to behavioral changes and clinical signs observed in zebrafish injected with tick saliva and then fed on mammal meat. The analysis suggests mast cell activation and the inhibition of pathways related to adrenergic signaling in cardiomyocytes, as well as heart and muscle contraction, supporting multisystemic organ involvement and linking alpha‐Gal sensitization to other illnesses. As in zebrafish mode, most dysregulated pathways in AGS patients following meat challenge are connected to lipid and fatty acid metabolism, potentially impacting nutrient absorption and microbiome composition	Vaz‐Rodrigues et al. [106]
Quantitative analysis of *I. ricinus* tick salivary proteome and lipidome, characterized by low glycan content, revealed an association between tick bites and the production of phosphatidylcholine‐IgG antibodies. Additionally, diacylglycerol levels were significantly higher in tick salivary glands compared to saliva and different salivary fractions. These findings support a potential role for tick salivary proteins and lipids without alpha‐Gal modifications in the AGS	Vaz‐Rodrigues et al. [107]
Metagenomics analysis of zebrafish gut microbiota composition in response to *I. ricinus* tick saliva and different tick salivary fractions, combined with alpha‐Gal‐positive dog food feeding, resulted in specific variations in microbiota composition at various taxonomic levels and affected alpha and beta diversities. This study revealed a potential role for gut microbiome components, including phyla Cyanobacteria, Firmicutes, Fusobacteriota, and Proteobacteria, in allergic reactions to mammalian meat consumption in AGS	Díaz‐Sánchez et al. [108]

The use of α1,3GalT‐KO mice in AGS studies has provided valuable insights into tick species‐specific roles, molecular and immunological mechanisms involved in sensitization, and the development of potential immunotherapeutic approaches. Notably, the results of the experiments in the α1,3GalT‐KO mouse model (Table 1) provided information on: (a) the production of total and alpha‐Gal‐specific IgE and IgG antibodies by sensitized mice in response to tick saliva; (b) how alpha‐Gal sensitization occurs only with certain tick species such as *A. americanum* , *A. sculptum* , and *I. ricinus* ; (c) the development of hypersensitivity and allergic reactions in mice sensitized with tick proteins and salivary gland extracts following an oral mammalian meat challenge; (d) the identification of AGS immune mechanisms associated with the induction of Th2 CD4^+^ T cell responses, which enhance the production of anti‐alpha‐Gal sIgE antibodies, cytokines like IL‐4, and activate the B cell‐intrinsic MyD88/TLR signaling pathway; and (e) proposed immunotherapies, such as poly‐L‐lysine‐based alpha‐Gal‐glycoconjugates or biodegradable nanoparticles encapsulating alpha‐Gal, for the treatment of AGS‐related allergic reactions or to enhance immune tolerance against alpha‐Gal sensitization.

In the zebrafish model (Table 2), the results showed (a) the production of anti‐alpha‐Gal IgM antibodies, accompanied by the development of allergic reactions, abnormal behavior, and changes in feeding patterns in response to tick saliva and salivary biogenic substances following mammalian meat consumption; (b) allergic reactions were associated with tissue‐specific TLR‐mediated response, B cells, basophils, and cytokine production; (c) the inhibition of pathways associated with adrenergic signaling in cardiomyocytes, as well as heart and muscle contraction in response to alpha‐Gal sensitization; and (d) a role for tick salivary proteins and lipids without alpha‐Gal modifications in AGS, as well as the influence of the host gut microbiome composition on this disease.

Additionally, a recent study published in 2025 [112] showed that tick salivary proteins antigen p23 and metalloprotease, previously identified by Contreras et al. [105], are involved in zebrafish allergic symptomatology after mammalian meat consumption, driven by pro‐inflammatory inducers (DNA‐dependent protein kinase [DNA‐PK] and TLR2) and cytokines (tumor necrosis factor‐alpha [TNF‐α] and IL‐1β). The *M. marinum* challenge triggered a Th1‐driven protective response, diminishing Th2 allergic AGS‐related symptoms in zebrafish inoculated with tick salivary components and fed mammalian meat. Moreover, recombinant metalloprotease and antigen p23 proteins lowered *M. marinum* gut infection levels by promoting protective anti‐alpha‐Gal IgM antibody levels and reducing the expression of multiple pro‐inflammatory cytokines and mediators.

**FIGURE 2:**
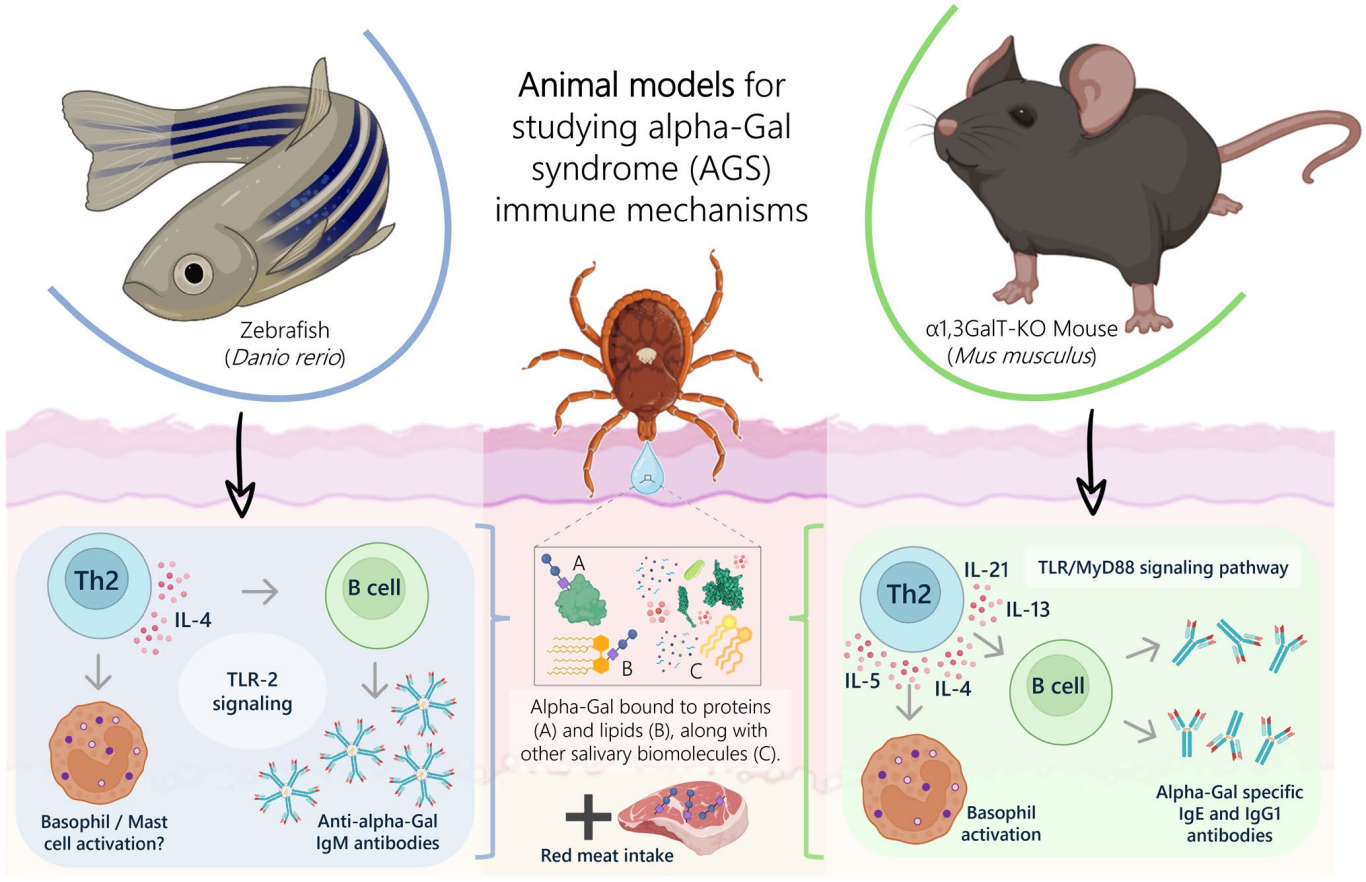
Key immune mechanisms altered in response to several tick saliva components, followed by mammalian meat consumption, in α1,3GalT‐KO C57BL/6 mouse ( *Mus musculus* ) and zebrafish ( *Danio rerio* ) models.

Taken together, these results provided evidence sustained by both animal models, including the production of anti‐alpha‐Gal antibodies in response to tick saliva, the development of allergic reactions in animals sensitized with tick protein extracts following mammalian meat consumption, and the identification of immune mechanisms associated with a skewed type 2 immune response, triggering TLR signaling, IL‐4 production, and humoral activity. Furthermore, the unique contributions of each animal model to the study of AGS may offer additional insights into the mechanisms associated with disease and protective responses (Figure 2, Table 3).

**FIGURE 3:**
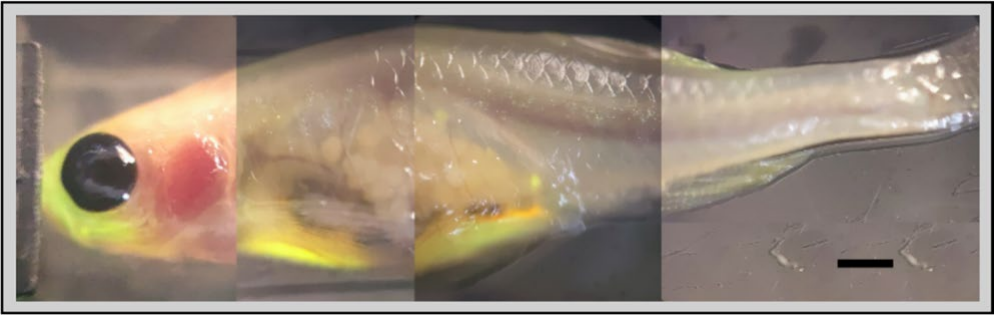
Fluorescent and confocal stacked images of GFP‐labeled adult Casper strain of zebrafish ( *Danio rerio* ) demonstrating the ability to visualize internal organs through the fish's skin (scale bar = 1 mm).

**TABLE 3:** Summary of results from α1,3GalT‐KO mice and zebrafish models in AGS studies.

Results from α1,3GalT‐KO mice (Table 1)	Results from zebrafish model (Table 2)
Response to tick biomolecules	
Production of total and tick‐specific IgEs in response to tick protein extracts followed by increased anti‐alpha‐Gal sIgE levels and hypersensitivity reactions to mammalian meat consumption	Presence of anti‐alpha‐Gal IgM antibodies, allergic reactions, abnormal behavior, and feeding patterns in response to tick salivary components Potential role for tick salivary proteins and lipids (e.g., diacylglycerol) without alpha‐Gal modifications
Immune mechanisms	
Immune mechanisms associated with IgE response included cognate CD4^+^ T cells and B cell‐intrinsic Toll‐like receptor (TLR) adaptor protein MyD88. A higher number of basophils at the sensitization site, as well as alpha‐Gal‐specific B cells, germinal center B cells, and B cells of the IgE and IgG1 isotypes in skin‐draining lymph nodes. Systemic anaphylaxis, dependent on IL‐4, when challenged with alpha‐Gal‐containing glycoproteins and glycolipids	Allergic reactions were associated with tissue‐specific TLR‐mediated responses in Th1 and Th2 cells with a potential role for basophils in response to tick saliva Protective immune mechanisms included B‐cell maturation, antibody‐mediated opsonization of mycobacteria, FcR‐mediated phagocytosis, macrophage response, interference with the alpha‐Gal antagonistic effect of the TLR2/NF‐kB‐mediated immune response, and upregulation of pro‐inflammatory cytokines such as IL‐1β Modulation of immune response to alpha‐Gal by p23 and metalloprotease among other tick salivary proteins Potential role for gut microbiome components, including phyla Cyanobacteria, Firmicutes, Fusobacteriota, and Proteobacteria, in allergic reactions to mammalian meat consumption in AGS
Potential immunotherapies	
Immunotherapy based on poly‐L‐lysine‐based alpha‐Gal‐glycoconjugates for potential treatment of allergic disorders like AGS by selectively inhibiting the production of anti‐alpha‐Gal IgE antibodies Alpha‐Gal‐containing nanoparticles for increasing alpha‐Gal‐specific immune tolerance through modifications of immune mechanisms associated with alpha‐Gal‐induced IgE‐mediated allergic reactions	Protective response against mycobacteriosis by probiotic bacteria with high alpha‐Gal content

## 4 Study of Alpha‐Gal‐Associated Immune Protective Mechanisms

Protective immune mechanisms associated with allergic reactions in the AGS have been linked to the depletion of IL‐4 in α1,3GalT‐KO mice [59] and a Th1‐mediated response in the zebrafish model [109, 112]. Additionally, cross‐pathogen protective mechanisms mediated by trained immunity in the zebrafish model are potentially connected with alpha‐Gal [111]. The polyreactive protective capacity of anti‐alpha‐Gal IgM and IgG antibodies in humans [20, 113] has been proposed to be implicated in diseases such as malaria [114, 115, 116], Chagas disease [117], leishmaniasis [118, 119], tuberculosis [114, 116, 120], and zoonotic viral infections [121, 122]. Similar findings have been observed in α1,3GalT‐KO mice for *Trypanosoma* spp. [123], *Leishmania* spp. [124, 125], and *Plasmodium* spp. [126] infections, as well as in the zebrafish animal model for *Mycobacterium* spp. [109, 110]. These results support the use of both mouse and zebrafish animal models to study the protective capacity of the immune response to alpha‐Gal against infectious and parasitic diseases.

## 5 Limitations and Concerns of the Zebrafish Model for the AGS Study

Despite these results, limitations and concerns have been considered and addressed for the zebrafish animal model in the study of the AGS (Table 4).

**TABLE 4:** Addressing limitations and concerns for the zebrafish ( *Danio rerio* ) model in AGS.

Proposed limitations and concerns	Addressing directions
Rather than being a model of an allergic hypersensitivity response, zebrafish resembles a model of a toxin‐induced response Zebrafish is rather a toxigenic model to tick saliva rather than a model of an allergic disease	To mimic exposure to tick saliva, zebrafish are first sensitized with tick saliva on day one. On day two, they start oral exposure to dog food (alpha‐Gal positive), and on the third day, they are treated again with tick saliva. This sequence of treatments is designed to reproduce the development of allergic reactions typically seen in response to tick saliva and mammalian meat exposure Treatment with tick saliva, but not alpha‐Gal alone, induces allergic reactions and mortality in zebrafish fed on dog food (alpha‐Gal positive) but not on fish feed (alpha‐Gal negative). This suggests that tick saliva triggers generalized allergic responses specifically when combined with the presence of alpha‐Gal in the diet
Injected tick saliva intramuscularly instead of into the collagenous stroma or the epidermis as the equivalent of the skin of the zebrafish	Zebrafish are injected with tick saliva by inserting the needle 2 mm underneath the skin holding the syringe at an angle of 15°–20° to the horizontal plane near the fish tail to mimic tick bites. IM injection ensures a controlled and reproducible administration of saliva. Although this approach does not perfectly replicate the natural tick bite, it was a necessary adaptation that still mimics the host immune response to tick saliva as closely as possible
Identified pathways are associated with cardiomyocyte function and muscle contraction. Is it because fish are dying from toxic shock after tick saliva injection and dog food feeding (alpha‐Gal positive), rather than an allergic reaction?	Studies utilizing integrated multi‐omics datasets, including metagenomics, lipidomics and proteomics, in zebrafish treated with tick saliva and deglycosylated saliva fraction suggest that the observed effects on cardiomyocyte function and muscle contraction were linked to allergic reactions, not toxic shock. The deglycosylated saliva fraction acts as a control because the alpha‐Gal epitopes were enzymatically removed. Zebrafish exposed to this salivary fraction and fed on alpha‐Gal positive dog food showed significantly reduced allergic responses compared to those exposed to intact tick saliva. This finding indicates that the immune reaction to the glycan moieties, rather than toxic shock after tick saliva injection and alpha‐Gal‐positive dog food feeding, is responsible for the observed effect
Teleost fish do not produce IgE, making zebrafish an unsuitable model for studying AGS mechanisms	The results from multiple studies support the use of the zebrafish model for studying AGS, including the production of anti‐alpha‐Gal IgM antibodies, the development of allergic reactions, abnormal behavior and altered feeding patterns in response to tick saliva, salivary biogenic substances, and mammalian meat consumption. Zebrafish studies highlight the association of allergic reactions with tissue‐specific TLR‐mediated response, IL‐4 and basophils; the inhibition of pathways related to adrenergic signaling in cardiomyocytes, and heart and muscle contraction in response to alpha‐Gal sensitization, and the role of tick salivary proteins and lipids without alpha‐Gal modifications in AGS. In contrast to the antibody isotypes found in humans and mice (IgD, IgM, IgA, IgG and IgE), zebrafish produce only functionally equivalent IgZ/IgT associated with mucosal immunity, along with IgM and IgD for the other immune functions
When immunized with protein xenoantigens (tick to fish), Th2 responses are induced as the normal immune response. When Th1 responses are enhanced by infection with a microorganism such as *Mycobacterium marinum* , Th2 responses are normally suppressed. Therefore, these findings do not provide a new molecular mechanism for the immune response induced by alpha‐Gal	The infection with *M. marinum* activated Th1‐mediated protective mechanisms, reducing Th2 allergic AGS‐related symptoms in zebrafish inoculated with tick salivary components and fed on mammalian meat. However, metalloprotease and antigen p23 tick salivary proteins reduced *M. marinum* gut infection levels and the risk of tuberculosis through protective anti‐alpha‐Gal IgM antibodies and lowered expression of pro‐inflammatory cytokines and mediators (TNF‐α, IL‐1β, TLR2, and DNA‐PK)
Organs critical for immune and allergic responses to alpha‐Gal are absent in zebrafish. In contrast, mice possess a more complex immune system that closely mirrors human immune responses, making them a more suitable model for studying AGS and related allergic mechanisms	In zebrafish, kidney and intestine are the key organs associated with the response to alpha‐Gal. The intestine is involved in both innate and adaptive immune responses. As stated in Contreras et al. [103], “in the intestine a mechanism similar to that proposed in humans and mouse model may trigger AGS through activation of TLR by alpha‐Gal leading to production of pro‐inflammatory cytokines and anti–alpha‐Gal IgE response. As proposed for humans in response to tick saliva, basophils in zebrafish may also be recruited to attract Th2 cells producing IL‐4 to the muscle inducing Th2‐mediated IgE response to alpha‐Gal”. Additionally, zebrafish are easier to handle in larger numbers and are more feasible for visualization (Figure 3)

## 6 Conclusions

The results obtained from multiple studies, along with responses to proposed limitations and concerns, support the suitability of zebrafish as a model for studying allergic reactions associated with tick saliva biomolecules and mammalian meat consumption, as observed in AGS. Similar results obtained in mouse and zebrafish models, together with their complementary contributions, support the use of both models rather than a single one for a more comprehensive characterization of AGS‐associated immune mechanisms. A similar conclusion was recently drawn in a Research Topic addressing animal models of allergic diseases, emphasizing the unique contributions of different animal models in understanding sensitization mechanisms and evaluating the potential therapeutic interventions in preclinical settings [127].

Future directions involve a deeper characterization of the immunological and allergenic pathways connected to the AGS, as well as the potential protective effects of various tick salivary proteins and other lipidome/metabolome biomolecules against infectious and parasitic diseases. This could potentially be done through in vitro studies using human basophils, mastocytes, or other relevant Th2‐activated cell types, as well as in vivo studies employing both mouse and zebrafish models, to better understand the molecular mechanisms underlying AGS. The zebrafish model has important utility in dissecting the immune pathways, which are an important functional component in infectious diseases and the AGS. Recent review papers have outlined the possibility of characterizing the molecular pathways using genetic analysis in zebrafish [128, 129, 130]. Furthermore, mutant and knockdown screening using CRISPR‐Cas9‐mediated mutagenesis may be used for characterization and understanding AGS [131]. The inactivation of key molecules identified in multiple research models used for the study of AGS [129] may be genetically manipulated in the zebrafish model to advance in the characterization, diagnosis, prevention, and treatment of AGS.

The integration of these results into dynamic datasets and comprehensive databases will significantly enhance our understanding of the AGS, contributing to the development of targeted prevention strategies, therapeutical interventions, and improved control measures. Accordingly, we conclude that the answer to the question posed in this study, “Is zebrafish a good model for the alpha‐Gal syndrome?” is yes, considering the addressed limitations and in combination with the mouse model.

## Author Contributions

Both authors contributed to writing and editing the manuscript and approved the final version.

## Conflicts of Interest

The authors declare no conflicts of interest.

## Data Availability

Data sharing is not applicable to this article as no datasets were generated or analyzed during the current study.
